# Supplementary material for: A fault-intrusion-tolerant system and deadline-aware algorithm for scheduling scientific workflow in the cloud
Source: PeerJ Comput Sci. 2021 Nov 2;7:e747. doi: 10.7717/peerj-cs.747 (PMC8576554; doi:10.7717/peerj-cs.747)
Supplement: Supplemental Information 6 [file peerj-cs-07-747-s006.zip › Myproject1.1/docs/org/cloudbus/cloudsim/core/CloudInformationService.html]

CloudInformationService


JavaScript is disabled on your browser.


- Overview
- Package
- Class
- Use
- Tree
- Deprecated
- Index
- Help

- Prev Class
- Next Class

- Frames
- No Frames

- All Classes

- Summary:
- Nested |
- Field |
- Constr |
- Method

- Detail:
- Field |
- Constr |
- Method


org.cloudbus.cloudsim.core

## Class CloudInformationService

- java.lang.Object
- - org.cloudbus.cloudsim.core.SimEntity
  - - org.cloudbus.cloudsim.core.CloudInformationService

- All Implemented Interfaces:
  :   java.lang.Cloneable

  ---

    

  ```
  public class CloudInformationService
  extends SimEntity
  ```

  A Cloud Information Service (CIS) is an entity that provides cloud resource registration,
  indexing and discovery services. The Cloud hostList tell their readiness to process Cloudlets by
  registering themselves with this entity. Other entities such as the resource broker can contact
  this class for resource discovery service, which returns a list of registered resource IDs. In
  summary, it acts like a yellow page service. This class will be created by CloudSim upon
  initialisation of the simulation. Hence, do not need to worry about creating an object of this
  class.

  Since:
  :   CloudSim Toolkit 1.0

- - ### Field Summary

    - ### Fields inherited from class org.cloudbus.cloudsim.core.SimEntity

      `FINISHED, HOLDING, RUNNABLE, WAITING`
  - ### Constructor Summary

    Constructors

    Constructor and Description

    `CloudInformationService(java.lang.String name)`

    Allocates a new CloudInformationService object.
  - ### Method Summary

    Methods

    | Modifier and Type | Method and Description |
    | --- | --- |
    | `java.util.List<java.lang.Integer>` | `getAdvReservList()` Gets the list of CloudResource IDs that **only** support Advanced Reservation. |
    | `java.util.List<java.lang.Integer>` | `getList()` Gets the list of all CloudResource IDs, including hostList that support Advance Reservation. |
    | `protected void` | `processEndSimulation()` Notifies the registered entities about the end of simulation. |
    | `void` | `processEvent(SimEvent ev)` Processes events scheduled for this entity. |
    | `protected void` | `processOtherEvent(SimEvent ev)` This method needs to override by a child class for processing other events. |
    | `boolean` | `resourceExist(int id)` Checks whether the given CloudResource ID exists or not. |
    | `boolean` | `resourceExist(java.lang.Integer id)` Checks whether the given CloudResource ID exists or not. |
    | `boolean` | `resourceSupportAR(int id)` Checks whether a given resource ID supports Advanced Reservations or not. |
    | `boolean` | `resourceSupportAR(java.lang.Integer id)` Checks whether a given resource ID supports Advanced Reservations or not. |
    | `void` | `shutdownEntity()` Shutdowns the CloudInformationService. |
    | `protected void` | `signalShutdown(java.util.Collection<java.lang.Integer> list)` Sends a signal to all entity IDs mentioned in the given list. |
    | `void` | `startEntity()` Starts the CloudInformationService entity. |

    - ### Methods inherited from class org.cloudbus.cloudsim.core.SimEntity

      `cancelEvent, clone, getEventBuffer, getId, getName, getNextEvent, getNextEvent, getState, numEventsWaiting, numEventsWaiting, pause, run, schedule, schedule, schedule, schedule, scheduleFirst, scheduleFirst, scheduleFirst, scheduleFirst, scheduleFirstNow, scheduleFirstNow, scheduleFirstNow, scheduleFirstNow, scheduleNow, scheduleNow, scheduleNow, scheduleNow, selectEvent, send, send, send, send, sendNow, sendNow, sendNow, sendNow, setEventBuffer, setId, setState, waitForEvent`
    - ### Methods inherited from class java.lang.Object

      `equals, finalize, getClass, hashCode, notify, notifyAll, toString, wait, wait, wait`

- - ### Constructor Detail


    - #### CloudInformationService

      ```
      public CloudInformationService(java.lang.String name)
                              throws java.lang.Exception
      ```

      Allocates a new CloudInformationService object.

      Parameters:
      :   `name` - the name to be associated with this entity (as required by SimEntity class)

      Throws:
      :   `java.lang.Exception` - This happens when creating this entity before initialising CloudSim package
          or this entity name is null or empty
  - ### Method Detail


    - #### startEntity

      ```
      public void startEntity()
      ```

      Starts the CloudInformationService entity.

      **Specified by:**
      :   `startEntity` in class `SimEntity`


    - #### processEvent

      ```
      public void processEvent(SimEvent ev)
      ```

      Processes events scheduled for this entity.

      **Specified by:**
      :   `processEvent` in class `SimEntity`

      Parameters:
      :   `ev` - the event to be handled.

      See Also:
      :   `SimEntity.processEvent(SimEvent)`


    - #### shutdownEntity

      ```
      public void shutdownEntity()
      ```

      Shutdowns the CloudInformationService.

      **Specified by:**
      :   `shutdownEntity` in class `SimEntity`


    - #### getList

      ```
      public java.util.List<java.lang.Integer> getList()
      ```

      Gets the list of all CloudResource IDs, including hostList that support Advance Reservation.

      Returns:
      :   LinkedList containing resource IDs. Each ID is represented by an Integer object.


    - #### getAdvReservList

      ```
      public java.util.List<java.lang.Integer> getAdvReservList()
      ```

      Gets the list of CloudResource IDs that **only** support Advanced Reservation.

      Returns:
      :   LinkedList containing resource IDs. Each ID is represented by an Integer object.


    - #### resourceSupportAR

      ```
      public boolean resourceSupportAR(java.lang.Integer id)
      ```

      Checks whether a given resource ID supports Advanced Reservations or not.

      Parameters:
      :   `id` - a resource ID

      Returns:
      :   true if this resource supports Advanced Reservations, false
          otherwise


    - #### resourceSupportAR

      ```
      public boolean resourceSupportAR(int id)
      ```

      Checks whether a given resource ID supports Advanced Reservations or not.

      Parameters:
      :   `id` - a resource ID

      Returns:
      :   true if this resource supports Advanced Reservations, false
          otherwise


    - #### resourceExist

      ```
      public boolean resourceExist(int id)
      ```

      Checks whether the given CloudResource ID exists or not.

      Parameters:
      :   `id` - a CloudResource id

      Returns:
      :   true if the given ID exists, false otherwise


    - #### resourceExist

      ```
      public boolean resourceExist(java.lang.Integer id)
      ```

      Checks whether the given CloudResource ID exists or not.

      Parameters:
      :   `id` - a CloudResource id

      Returns:
      :   true if the given ID exists, false otherwise


    - #### processOtherEvent

      ```
      protected void processOtherEvent(SimEvent ev)
      ```

      This method needs to override by a child class for processing other events. These events are
      based on tags that are not mentioned in `#body()` method.

      Parameters:
      :   `ev` - a Sim\_event object


    - #### processEndSimulation

      ```
      protected void processEndSimulation()
      ```

      Notifies the registered entities about the end of simulation. This method should be
      overridden by the child class


    - #### signalShutdown

      ```
      protected void signalShutdown(java.util.Collection<java.lang.Integer> list)
      ```

      Sends a signal to all entity IDs mentioned in the given list.

      Parameters:
      :   `list` - List storing entity IDs


- Overview
- Package
- Class
- Use
- Tree
- Deprecated
- Index
- Help

- Prev Class
- Next Class

- Frames
- No Frames

- All Classes

- Summary:
- Nested |
- Field |
- Constr |
- Method

- Detail:
- Field |
- Constr |
- Method
